# Cyclin-dependent kinases regulate the adult nervous system via the one-carbon-metabolism

**DOI:** 10.1038/s41419-023-05950-6

**Published:** 2023-07-14

**Authors:** Alessia Angelin

**Affiliations:** Center for Mitochondrial and Epigenomic Medicine, Division of Human Genetics, The Children’s Hospital of Philadelphia, Philadelphia, PA USA

**Keywords:** Kinases, Cell death in the nervous system

Cyclin-dependent kinases (CDKs) are members of the serine/threonine protein kinase family present in all eukaryotic cells, activated by binding proteins as cyclins. CDKs were originally discovered for their role in regulating cell cycle, and subsequently, found to modulate transcription, mRNA processing, DNA repair and differentiation of nerve cells, as well as orchestrating cytoskeleton dynamics [1]. The human genome encodes a total of 21 CDKs, which, according to the different cellular processes regulated by CDKs, are divided into two main subfamilies: cell cycle CDKs (CDK1-6, -11, -14–18) and transcriptional CDKs (CDK7-13, -19, -20) [2]. Given their fundamental role in cell cycle regulation, it is not surprising that numerous studies have demonstrated their importance in tumorigenesis, in particular dysregulation of CDKs expression due to genetic mutations or impaired epigenetic regulation has been linked to tumor progression [3]. A relevant example is CDK12, which, by binding its cyclin chaperone cyclin K, promotes phosphorylation of RNA polymerase II and modulates transcriptional elongation in human and Drosophila. Mutations or deletions of *CDK12* have been found in cancers, leading to specific pharmacological approaches [2, 4]. Moreover, CDK12 has been shown to be relevant for mouse embryo neuronal development. Knockdown of CDK12 results in a decrease of numbers of neurons, and in primary culture cortical neurons shortens the averaged axonal length. Interestingly, CDK12 mutant mice showed a decrease of CDK5 expression in the developing mouse brain, which correlates with a reduced axonal outgrowth, while in-vitro overexpression of CDK5 partially rescues the defect observed when CDK12 is depleted. This suggests that CDK5 could be involved in mediating the effect of CDK12 on axonal elongation [5]. In Drosophila, Pan et al. showed that CDK12 modulates heterochromatin enrichment in several chromosomes and reduces the transcription of multiple neuronal genes in the adult brain, thus resulting in a defect in courtship learning. They identified a role for CDK12 in controlling the epigenetic transition between euchromatin and heterochromatin, which suggests a chromatin regulatory mechanism relevant to neuronal behavior [6]. Now, CDK12 regulates the adult nervous system via the one-carbon metabolism.

Recently, Townsend et al. [7] discovered a novel function of CDK12 in Drosophila adult neurons. Specifically, CDK12-/- neurons displayed a specific age-dependent swelling of the proximal axon region with the formation of large axonal blebs followed by neuronal degeneration. Interestingly, these axonal blebs were found associated with β-actin patches. Actin is normally present in small distinct patches in the axon proximal to the cell body region and its size and location undergo minimal changes during aging. The ablation of CDK12 induced a significant enlargement of β-actin patches at young ages, whereas an increase in their frequency at later stages. A subsequent evaluation of the actin filaments in Drosophila neurons showed that F-actin is enriched in the axon-soma boundary and that loss of CDK12 induced an increase in F-actin formation, particularly in the proximal axonal region. A prior study [8] revealed that the proximal axon is normally enriched in short and stable F-actin and is endowed with longer dynamic F-actin species, likely needed for diffusion barrier formation between the axon and the cell body. It is plausible that actin enrichment in the proximal axon and somato-dendritic regions might favor localization of ion channels and specific proteins, modulate myosin-dependent trafficking, and contribute to vesicle/organelle barrier formation. The available evidence suggests alternative models that could explain the organization of the axon initial segment (AIS), and one of them indicates that actin filaments are key components of a "fence" that limits the mobility of membrane proteins between adjacent “actin rings” [9]. Townsend et al. [7] also found that CDK12 exclusively localized to the nucleus of adult neurons, as opposed to CDK5 which was found in the cytoplasm, particularly in the AIS, although not associated with the blebs. The separate cell compartmentalization of CDKs in neurons could suggest different intracellular mechanisms with a combined effect in regulating axonal width and length. Given the localization of CDK12 in the nucleus, Townsend et al. [7] explored the possibility that actin re-organization may also be mediated by transcription regulation. By RNA sequencing analysis, they found several transcriptionally repressed genes associated with the folate pathway. These enzymes are not only critical for amino acid homeostasis, but also for modulating the homocysteine (hCYS) pool, see figure. Analysis of adult fly brains has revealed a significant increase in hCYS levels, known to be involved in the reorganization of actin cytoskeleton. To support their hypothesis, Townsend et al. [7] induced an increase of hCYS in human iPSC-derived cortical neurons and showed a sequential F-actin disorganization in neuronal projections and around the soma, including blebs and patches formation. Their results suggest that CDK12 normally regulates protein expression of the folate pathway to prevent hyperhomocysteinemia in neurons, and this maintains the F-actin filaments organization and limits neurodegeneration. This work is of crucial relevance, as it sheds new light into the role of CDKs in the adult nervous system; however, it is important to point out that the CDK12 is not the only CDK modulating the cytoskeleton in the brain. CDK5 was found essential for brain development during embryogenesis, but also in numerous neuronal processes in the mature brain, from neuronal plasticity to memory formation and pain signaling, which all rely on rapid alterations of the cytoskeleton [10]. Suppression of CDK5 activity leads to an overstabilization of microtubules and decreases microtubular dynamics, and this microtubule dysregulation disturbs cortical neuronal migration [11]. Furthermore, CDK5 phosphorylates many microtubule-associated proteins, such as Nudel, a dynein-interacting protein. Indeed, a mutation in Nudel that prevents CDK5 phosphorylation produces prominent axonal swelling in cultured neurons, as it occurs when Lis1 (Nudel dynein-interacting protein) or dynein are disrupted in Drosophila neurons [12]. Although many studies focus on the association between CDK5 and microtubules, CDK5 also colocalizes with F-actin in the growth cone of cultured neurons, and p39 and p35 (activators of CDK5 upon association) are reported to interact with F-actin [11]. The complex CDK5 / p27kip1 promotes the activity of the actin-binding protein Cofilin, suppresses RhoA and its downstream kinase, Rho kinase/ROCK, modulating cortical neuronal migration [10–12]. Additional CDK5 targets are Neurabin-I, a neuron-specific actin-binding protein which induces changes in neuronal morphologies by F-actin organization, and Drebrin, a F-actin-binding protein that regulates neurogenesis and spine morphologies [10, 11]. Townsend et al. [7] did not investigate the possibility that CDK12 could also modulate CDK5 and contribute to the F-actin organization through multiple mechanisms.

CDK may have distinct functions in Drosophila and mammalian cells [13], and their interaction with the cytoskeleton, particularly actin filaments, is important for neuronal cells as evident in multiple neurological disorders. Indeed, Alzheimer, Parkinson, Amyotrophic lateral sclerosis, and Down’s syndrome all exhibit profound cytoskeletal abnormalities; the same applies to several memory disorders [10, 12]. Accordingly, elevated hCYS is a risk factor for age-dependent neurological conditions, where hCYS levels correlate with disease progression [14]. Thus, Townsend et al. [7] have provided a novel molecular pathway to link together neuronal pathological events (Fig. 1).

**Fig. 1:**
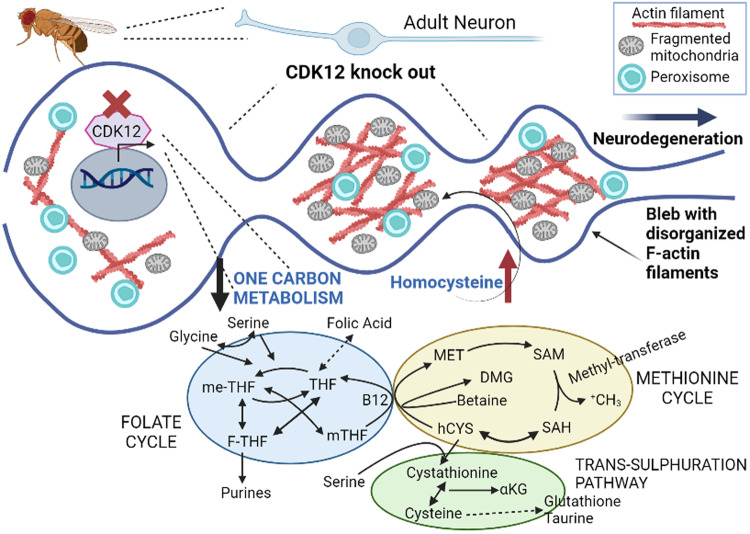
Cyclin-dependent kinases regulate the adult nervous system via the one-carbon metabolism. Knockout CDK12 directly affects the formation of the adult nervous system, see for details Townsend et al. [7], via the one-carbon-metabolism. the one-carbon-metabolism consists of two modules, the folate and the methionine cycles. Folic acid is imported into cells and reduced to tetrahydrofolate (THF), before being converted to 5,10-methylene-THF (me-THF) by serine-hydroxymethyl-transferase (SHMT), and then reduced to 5-methyl-THF (mTHF) by methylene-THF-reductase (MTHFR) or converted into 10-formyl-THF (F-THF). mTHF is demethylated to close the folate cycle, and, more importantly, donating one carbon into the methyonine cycle via the methylation of homocysteine (hCYS) by the methionine synthase that uses vitamine B12 as cofactor, thus forming methionine (MET). MET, via the MET-adenyltransferase (MAT), generates S-adenosylmethionine (SAM), which, in turn, is demethylated into S-adenosylhomocysteine (SAH). Then, SAH-hydrolase (SAHH), converts SAH into hCYS, closing the cycle. The one-carbon-metabolism can be connected to the trans-sulfuration pathway, generating cystathionine, and, in turn, either a-ketobutyrate (aKB) or glutathione. (Created with Biorender.com).

Last, but not least, cytoskeleton, and actin filaments, are relevant for mitochondrial network maintenance. The actin changes associated with loss of CDK12 impact mitochondrial dynamics resulting in an increase of fragmented mitochondria abundant in axonal swellings. The authors associated the changes in mitochondria to DRP1-mediated fission, although this aspect needs to be further explored in future studies. The putative involvement of DRP1 in this CDK12−/− model is intriguing because CDK12 mutant neurons also manifested alterations in peroxisome structure and localization. In fact, mitochondria and peroxisomes have an extensive crosstalk, including exchange of division machinery proteins. In Drosophila neurons peroxisomes are exclusively localized to the actin rich somato-dendritic regions, and the ablation of CDK12 caused peroxisomes structure abnormality and their entrance in the proximal axon in an age-dependent manner. This was also correlated with the formation of axonal actin patches. Hence, CDK12 regulates the “actin fence”, serving as an important vesicle/organelle barrier between the cell body and the axon. Townsend et al. [7] concluded that mitochondrial fragmentation occurs as a consequence of the F-actin disorganization, and that the neurodegeneration caused by ablation of CDK12 is likely due to heightening of actin formation rather than aberrant mitochondrial dynamics. However, we cannot exclude the possibility that mitochondria significantly contribute to the amplification of neuronal dysfunction. In fact, several studies indicate a complex bidirectional relationship between cell cycle regulators and mitochondria that must ensure the supply of sufficient fuel and metabolites for cell growth and survival. For instance, multiple CDKs have been found to be involved in mitochondria biogenesis and complex I activation by phosphorylation [13, 15]. Some CDKs have also been found to directly phosphorylate DRP1 and mediate mitochondrial fission [13], such as CDK5 in mitotic cells [15], and in rat post-mitotic mature neurons [13].

Further investigation on mitochondria contributing to neuronal degeneration in CDK-related disorders might foster novel therapies with mitochondria-targeted drugs.

## References

[1] Bendris N, Lemmers B, Blanchard JM (2015). Cell cycle, cytoskeleton dynamics and beyond: the many functions of cyclins and CDK inhibitors. Cell Cycle.

[2] Lei P, Zhang J, Liao P, Ren C, Wang J, Wang Y (2022). Current progress and novel strategies that target CDK12 for drug discovery. Eur J Med Chem.

[3] Ghafouri‑Fard S, Khoshbakht T, Hussen BM, Dong P, Gassler N, Taheri M (2022). A review on the role of cyclin dependent kinases in cancers. Cancer Cell Int.

[4] Wu W, Yu S, Yu X (2023). Transcription-associated cyclin-dependent kinase 12 (CDK12) as a potential target for cancer therapy. Biochim Biophys Acta Rev Cancer.

[5] Chen H, Lin GT, Huang CK, Fann MJ (2014). Cdk12 and Cdk13 regulate axonal elongation through a common signaling pathway that modulates Cdk5 expression. Exp Neurol.

[6] Pan L, Xieb W, Lic KL, Yanga Z, Xua J, Zhang W (2015). Heterochromatin remodeling by CDK12 contributes to learning in Drosophila. Proc Natl Acad Sci USA.

[7] Townsend L, H Clarke, D Maddison, KM Jones, L Amadio, A Jefferson, et al. Cdk12 maintains the integrity of adult axons by suppressing actin remodeling. Cell Death Discovery. 2023. https://ssrn.com/abstract=4239128. 10.1038/s41420-023-01642-4 PMC10511712 37730761

[8] Jones S, Korobova F, Svitkina T (2014). Axon initial segment cytoskeleton comprises a multiprotein submembranous coat containing sparse actin filaments. J Cell Biol.

[9] Huang Y, Rasband MN (2016). Organization of the axon initial segment: Actin like a fence. J Cell Biol.

[10] Shah K, Rossie S (2018). Tale of the good and the bad Cdk5: remodeling of the actin cytoskeleton in the brain. Mol Neurobiol.

[11] Kawauchi T (2014). Cdk5 regulates multiple cellular events in neural development, function and disease. Dev Growth Differ.

[12] Smith D, Tsai LH (2002). Cdk5 behind the wheel: a role in trafficking and transport?. Trends Cell Biol.

[13] Lopez Mejia I, Fajas L (2015). Cell cycle regulation of mitochondrial function. Curr Opin Cell Biol.

[14] Seshadri S, Beiser A, Selhub J, Jacques PF, Rosenberg IH, D’Agostino RB (2002). Plasma homocysteine as a risk factor for dementia and Alzheimer’s disease. N. Engl J Med.

[15] Huber K, Mestres-Arenas A, Fajas L, Leal-Esteban LC (2021). The multifaceted role of cell cycle regulators in the coordination of growth and metabolism. FEBS J.

